# Over‐the‐counter to under‐the‐skin: Expanding dermatology medical education

**DOI:** 10.1111/medu.15668

**Published:** 2025-03-06

**Authors:** Ellen Overson, Aakanksha Angra

## 1 WHAT PROBLEM WAS ADDRESSED?

Over‐the‐counter (OTC) medications are ubiquitous in the United States and despite being easily accessible, they pose potential health risks for consumers due to misuse, abuse, overdose, side effects and/or interactions with other medications. One of the most common skin conditions that dermatologists and non‐dermatologists treat is acne vulgaris and topical OTC products are typically recommended as first‐line treatments. Given that skin is a visible organ, doctors are often simultaneously treating medical and cosmetic concerns. If the doctors' recommendations fail to meet the patients' needs, patients often turn to what they see in stores: products which are a mix of topical medications and cosmeceuticals. Topical cosmeceuticals are skincare products with low concentrations of biologically active ingredients.^1^ Direct‐to‐consumer marketing and social media can make identifying facts about product safety and efficacy challenging, especially because the Food and Drug Administration (FDA) requires less safety and efficacy testing for a product that is designated a ‘cosmetic’ rather than a ‘drug’.^1^ Many doctors struggle to effectively guide patients in this field since OTC medications have been historically under‐emphasized in undergraduate medical education. To better equip medical students, we designed a hands‐on group project that challenged students to critically assess OTC product efficacy, safety, direct‐to‐consumer marketing and apply evidence‐based decision‐making in patient care.

## 2 WHAT WAS TRIED?

Our dermatology course was implemented concurrently across two geographically separate campuses of the medical school. Students were assigned to small groups and asked to select an active ingredient from a topical OTC product list. The list was created by the authors based on the active ingredients in popular topical products. Students were asked to create a poster to display what they learned about their product's mechanism of action, evidence of efficacy, recommended uses, precautions, contraindications, side effects and adverse reactions. A virtual poster session was held and students presented to each other. They were given a rubric developed by the authors and were asked to critique the content areas across three levels of assessment: present, needs improvement or absent.

## 3 WHAT LESSONS WERE LEARNED?

The project has been incorporated into the course for 2 years and despite this project only representing 10% of their final grade, it was completed by 100% of the students. Ninety five per cent of the students received full points on the assignment. Student feedback was overall positive, with many expressing that the project was an effective hands‐on, collaborative learning experience that fostered critical thinking and teamwork skills. This virtual format was an effective way to bring students from both campuses together which fostered community. On the constructive side, despite setting aside dedicated curricular time for project work, some students still found it to be more time‐consuming than expected and would have preferred learning the material via lecture instead. To better balance the time invested with the knowledge gained, we may consider simplifying the project to focus solely on evidence of efficacy and mechanism of action. We will continue to gather feedback to shape the project given the ongoing need for increased undergraduate education on OTC medications.

## AUTHOR CONTRIBUTIONS

**Ellen Overson:** Conceptualization; writing—original draft; writing—review and editing; project administration; supervision; methodology; software; data curation; formal analysis. **Aakanksha Angra:** Data curation; formal analysis; writing—original draft; writing—review and editing; methodology; software.

## CONFLICT OF INTEREST STATEMENT

The authors have no conflicts of interest to disclose.

## Data Availability

Our insights were gleaned from student feedback and individual responses will not be shared due to student privacy.
